# Translational aspects of cardiac cell therapy

**DOI:** 10.1111/jcmm.12632

**Published:** 2015-06-27

**Authors:** Cheng-Han Chen, Konstantina-Ioanna Sereti, Benjamin M Wu, Reza Ardehali

**Affiliations:** a Division of Cardiology, Department of Medicine, David Geffen School of Medicine at UCLALos Angeles, CA, USA; b Department of Bioengineering, UCLALos Angeles, CA, USA; c Eli and Edythe Broad Stem Cell Research Center, UCLALos Angeles, CA, USA

**Keywords:** cell therapy, cardiac progenitor cell, cardiac regeneration, direct reprogramming, combination cell therapy, biomaterials

## Abstract

Cell therapy has been intensely studied for over a decade as a potential treatment for ischaemic heart disease. While initial trials using skeletal myoblasts, bone marrow cells and peripheral blood stem cells showed promise in improving cardiac function, benefits were found to be short-lived likely related to limited survival and engraftment of the delivered cells. The discovery of putative cardiac ‘progenitor’ cells as well as the creation of induced pluripotent stem cells has led to the delivery of cells potentially capable of electromechanical integration into existing tissue. An alternative strategy involving either direct reprogramming of endogenous cardiac fibroblasts or stimulation of resident cardiomyocytes to regenerate new myocytes can potentially overcome the limitations of exogenous cell delivery. Complimentary approaches utilizing combination cell therapy and bioengineering techniques may be necessary to provide the proper milieu for clinically significant regeneration. Clinical trials employing bone marrow cells, mesenchymal stem cells and cardiac progenitor cells have demonstrated safety of catheter based cell delivery, with suggestion of limited improvement in ventricular function and reduction in infarct size. Ongoing trials are investigating potential benefits to outcome such as morbidity and mortality. These and future trials will clarify the optimal cell types and delivery conditions for therapeutic effect.

## Introduction

Ischaemic heart disease, in the form of acute myocardial infarction (MI) and resultant ischaemic cardiomyopathy, remains the leading cause of morbidity and mortality worldwide 1. Despite significant improvements in cardiac care over the past 50 years especially in primary and secondary prevention, approximately 1 million MIs still occur each year in the United States. Many of these patients go on to develop heart failure, which now affects over 5 million patients 2. While medications such as beta-blockers, angiotensin-converting enzyme inhibitors, and aldosterone antagonists can ameliorate decline in heart function, end-stage heart failure frequently necessitates complete or partial replacement of cardiac function with either heart transplant or a mechanical assist device 3.

With a MI, the heart can lose over a billion cells, approximately 25% of its mass 4. To compensate for the loss of cells, the affected area forms fibrotic scar tissue by activated fibroblasts and the immune response. Although tissue regeneration is a phenomenon occurring in adult mammalian tissues such as liver, skeletal muscle, bone and skin, the ability of the adult heart to renew itself is limited 5. This is not the case for lower vertebrates that are able to fully regenerate cardiac tissue following substantial injury 6. Until recently, the heart itself was thought to be a terminally differentiated organ. Bergmann *et al*. utilized the increased global levels of ^14^C from Cold War atomic bomb testing to date cardiomyocytes in patients who lived during that time period, and found evidence based on mathematical modelling for renewal of cardiomyocytes of up to 1% per year for a 20 year old 7. This renewal rate gradually decreased with age, to a yearly rate of 0.4% by age 75. Through these and other studies 8–10, it is now understood that endogenous repair mechanisms do exist in the adult mammalian heart, albeit at a capacity which is unable to fully counteract the damage caused by a MI.

Cardiac cell therapy, either through transplantation of exogenous cells or stimulation of endogenous resident cells, has been widely studied as a potential method for repair and regeneration of cardiac tissue. This manuscript explores the translational aspects of cardiac cell therapy, including cell source selection for exogenous delivery, strategies to regenerate cardiac tissue through direct reprogramming of endogenous cells, and enhancement of native cardiomyocyte proliferation through delivery of growth and transcription factors. We will also explore future directions in the field including combination cell therapy and bioengineering techniques.

## Exogenous cell delivery

**Figure 1:**
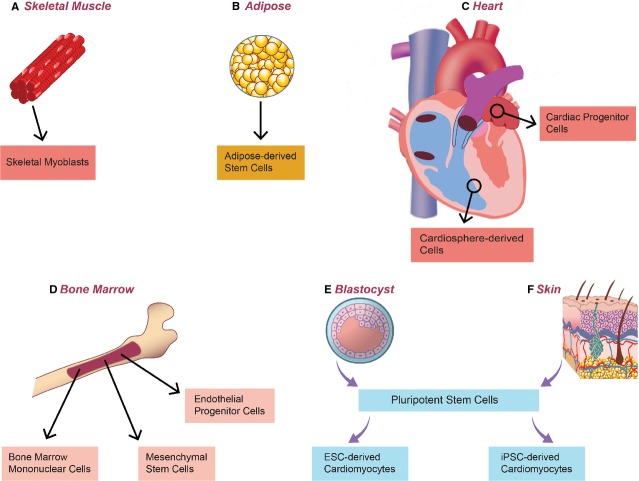
Cell and tissue sources of cells for exogenous cell delivery. Multiple clinical trials have investigated non-cardiac cells including (A) skeletal myoblasts, (B) adipose-derived stem cells, and (D) bone marrow-derived stem cells, with limited evidence of cell engraftment or clinical efficacy. Clinical trials utilizing cells obtained from biopsied cardiac tissue (C) including cardiac ‘progenitor’ cells and cardiosphere-derived cells have provided the strongest evidence to date for clinical efficacy of exogenous cell therapy. Embryonic stem cells (E) and induced pluripotent stem cells (F) can be used as a source of cardiomyocytes potentially capable of electromechanical integration into native cardiac tissue.

A wide variety of stem cell types have been evaluated for therapeutic delivery for cardiac repair, ranging from unipotent skeletal myoblasts to pluripotent embryonic stem cells (Figure1). Starting with early studies utilizing skeletal myoblasts and bone marrow stem cells, the rationale for stem cell delivery was predicated on the speculation that ‘plasticity’ of adult stem cells may lead to transdifferentiation of these cell types into cardiomyocytes to ‘regenerate’ native damaged tissue. Although it is now understood that the positive effects of cell delivery on cardiac function in these early studies may have resulted from a paracrine effect rather than true cell engraftment and differentiation into cardiomyocytes, multiple pre-clinical and clinical studies have been performed demonstrating relative safety and modest efficacy of these cell types. With the discovery of cardiac ‘progenitor’ cells as well as advancements in pluripotent stem cell (PSC) derivation, there is now the possibility for delivery of cardiomyocyte progenitors and cardiomyocytes capable of true engraftment and regeneration of cardiac tissue. Many questions remain with exogenous cell delivery techniques, including the choice of the best cell type for therapeutic effect as well as proper delivery method, given the low engraftment rates as well as the propensity for arrhythmogenesis.

### Skeletal myoblasts

Initial studies using a cell-based strategy for ischaemic heart disease relied on skeletal myoblasts, based on its ability to regenerate skeletal muscle through proliferation of quiescent satellite cells located under the basal lamina 11. Advantages of using this cell type include easy expansion *ex vivo*, and the ability to use an autologous source. Although preclinical studies demonstrated potential for intramyocardial injection of skeletal myoblasts to improve LV function likely through a mechanical scaffolding effect 12,13, multiple clinical trials including MAGIC 14 and MARVEL 15 have since revealed lack of efficacy when compared to placebo. Further studies showed that the injected cells do not integrate electromechanically with the surrounding myocardium (as they do not express connexin 43) 16, have a propensity to induce arrhythmias (especially dangerous ventricular tachyarrhythmias) 17, and do not regenerate myocardium 18. Considering lack of significant clinical improvement and their potential arrhythmogenic hazards, skeletal myoblasts have fallen out of favour as a therapeutic candidate.

### Bone marrow cells

As a result of decades of experience in the haematological realm for bone marrow transplants, bone marrow cells have been closely examined as a therapeutic option for cardiac cell therapy (Table1). These cells contain many inherent advantages, including ease in harvesting pure cell populations in large numbers, ability to be used allogeneically, and composition including fractions of stem and progenitor cells of different types. For these reasons, unselected bone marrow mononuclear cells have been the most widely tested in pre-clinical and clinical trials for cardiac therapy. Although an early study by Orlic *et al*. supported the idea that unselected bone marrow cells have the capability to differentiate into cardiomyocytes 8, this has since been discredited by a number of independent investigations 19,20. Several selected studies did, however, demonstrate improvement in cardiac function as well as decrease in infarct size 21,22. Other studies specifically examined the haematopoietic stem cell (HSC) subset of the unselected bone marrow population. Characterized by multiple distinct markers including CD133 and CD34 23. HSCs were shown in some pre-clinical studies to promote neovascularization and prevent LV remodelling 24. This subset of cells (accounting for less than 0.1% of unfractionated bone marrow mononuclear cells) may partially account for the positive effects of unselected bone marrow cell therapy 25.

**Table 1:** Notable cardiac cell therapy clinical trials recently completed or currently in progress

Study	Phase	Cell type	Population	Primary outcome(s)	Status/estimated completion
ALLSTAR 191	I/II	Allogeneic CDCs	Recent myocardial infarction with resultant ischaemic cardiomyopathy	Reduction in infarct size assessed by MRI	Recruiting/October 2015
RENEW 192	III	Autologous CD34^+^ EPCs	Chronic myocardial ischaemia	Improvement in total exercise time on Modified Bruce Protocol	Active, not recruiting/June 2016
PreSERVE-AMI 193	II	Autologous CD34^+^ HSCs	Acute ST-elevation myocardial infarction	Safety (measured by adverse events) and efficacy (measured by improvement in SPECT MPI)	Active, not recruiting/December 2016
AMICI 194	II	Allogeneic HSCs	Acute myocardial infarction	Safety (defined by major adverse cardiac and cerebral events)	Recruiting/June 2018
ADVANCE 195	II	Autologous ADCs	Acute ST-elevation myocardial infarction	Reduction in infarct size assessed by MRI	Completed
MyStromalCell 196	II	Autologous ADCs	Chronic ischaemic cardiomyopathy	Improvement in exercise test	Completed
ATHENA 197	II	Autologous ADCs	Chronic myocardial ischaemia	Safety (adverse events) and feasibility (change in mVO2, LVESV/LVEDV, EF and symptoms)	Active, not recruiting/May 2019
ATHENA II 198	II	Autologous ADCs	Chronic myocardial ischaemia	Improvement in symptoms (assessed by Minnesota Living with Heart Failure Questionnaire)	Active, not recruiting/May 2019
STEMI 199	II	Allogeneic MSCs	Acute ST-elevation myocardial infarction	Safety (by major adverse cardiac events)	Active, not recruiting/May 2017
STEM-104-M-CHF 200	II	Allogeneic MSCs	Chronic non-ischaemic cardiomyopathy	Safety	Recruiting/May 2015
Prochymal 201	II	Allogeneic MSCs	Acute myocardial infarction	Improvement in left ventricular end systolic volume	Active, not recruiting/February 2016

CDCs: cardiosphere-derived cells; EPCs: endothelial progenitor cells; HSCs: hematopoietic stem cells; ADCs: adipose-derived stem cells; MSCs: mesenchymal stem cells; MRI: magnetic resonance imaging; SPECT MPI: single photon emission computed tomography myocardial perfusion imaging; mVO2: mixed venous oxygen saturation; LVESV: left ventricular end systolic volume; LVEDV: left ventricular end diastolic volume; EF: ejection fraction.

Closely related to HSCs is the subset of circulating bone marrow mononuclear cells that are thought to specifically differentiate into endothelial cells. Endothelial progenitor cells (EPCs) were first characterized in 1997 by Asahara *et al*. 26 as expressing the HSC marker CD34 as well as an endothelial marker protein (most commonly VEGF-R2), and are thought to play a major role in neovascularization and maintenance of endothelial integrity under conditions of myocardial ischaemia 27. Initial animal studies using intramyocardial delivery of CD34^+^ cells in both rat 28 and porcine 29 models of MI showed promising improvements in cardiac function. These results led to several clinical studies specifically investigating cardiac transplantation of autologous CD34^+^ cells in chronic ischaemia (ACT34-CMI) 30, acute MI (TOPCARE-AMI) 31, and post-MI (TOPCARE-CHD) 32. All demonstrating safety of the therapy with some evidence of efficacy. The ongoing RENEW study will examine the efficacy of intramyocardial autologous CD34^+^ cell transplantation in patients with refractory angina 33.

More recently, it has been reported that there may be both ‘early’ and ‘late’ types of EPCs. Early EPCs are obtained from early 4–7 day *in vitro* cultures and express specific endothelial markers CD31 and TIE2, while late EPCs are cultured for at least 2–3 weeks *in vitro* and then express additional markers such as VE-cadherin and von Willebrand factor 34. It still remains unclear whether a specific EPC subset may promote substantial neovascularization in the injured myocardium, or whether the distinction exists purely *in vitro*.

Besides exogenous transplantation of bone marrow cells, a related therapeutic strategy has been the use of haematopoietic growth factors including granulocyte colony-stimulating factor 35, granulocyte macrophage colony-stimulating factor 36 and macrophage colony-stimulating factor 37 in the setting of myocardial injury. Their beneficial effect is predicated on mobilization of endogenous bone marrow stem cells including HSCs and EPCs which may then improve cardiac function through putative paracrine effects as well as a direct angiogenic effect on ischaemic tissue 38–40. Initial pre-clinical murine studies utilizing these factors demonstrated reduced LV remodelling as well as improved cardiac function 37,41,42. However, a number of pilot clinical trials have since shown variable outcome in terms of efficacy, with most of them unable to reproduce the favourable outcome seen in the animal studies 43–46.

A wide heterogeneity exists in the specific bone marrow cells used for the pre-clinical and clinical studies in this field, with differences in the cell isolation, storage and enrichment processes 47. The wide clinical experience with bone marrow cells for cardiac therapy has had mixed results, likely because of this heterogeneity. Ongoing trials with specific populations of purified bone marrow cells as described above will shed light on the promise of this cell type for future cardiovascular therapy.

### Mesenchymal stem cells

Another source for allogeneic cell therapy consists of mesenchymal stem cells (MSCs), also known as mesenchymal stromal cells or colony forming unit-fibroblasts. These were first isolated from bone marrow stroma and described by Friedenstein *et al*. 48 more than 40 years ago, and have been shown in the intervening decades to be a multi-potent source of mesoderm (as well as some non-mesoderm) derived tissues including osteoblasts, chondrocytes, adipocytes, skeletal muscle, hepatocytes and even neurons 49,50. The ability of MSCs to differentiate into cardiomyocytes is somewhat in dispute, however, with some studies demonstrating transdifferentiation of MSCs to cardiomyocytes 51,52 while many others showing very limited cardiomyogenic potential 53,54. Despite this controversy, MSCs have been eagerly pursued as a cell-based source for cardiac repair, because of their many other favourable properties including their immunomodulatory properties and their easy isolation and amplification from an allogeneic source 55.

The main role of the MSC is thought to be as a controller of stem cell niches, most importantly that of the HSCs in the bone marrow, but also in other tissues including the gut and hair follicles 56. There is no uniform definition for MSCs, but the International Society of Cell Therapy has proposed criteria for MSCs including: (*i*) the ability to adhere to plastic under normal culture conditions and display a fibroblast-like morphology, (*ii*) the ability to differentiate into osteoblasts, adipocytes, and chondrocytes *in vitro* and (*iii*) expression of surface markers CD73, CD90 and CD105, in the absence of CD11b, CD14, CD19, CD34, CD45, CD79a and HLA-DR (Human leukocyte antigen) 57.

Mesenchymal stem cells produce their immunomodulatory effects through their unique immunophenotype, the secretion of soluble factors, and through interactions with both the innate and adaptive immune cells. As they are negative for MHC II (Major Histocompatibility Complex), B7, and CD40, MSCs are tolerated well when allogeneically transplanted. By secreting factors such as interleukin-6, transforming growth factor (TGF)-β1, and prostaglandin E2, MSCs suppress innate immune cell inflammatory responses such as the respiratory burst function of neutrophils 58 and production of INF-γ (Interferon alpha) by natural killer cells 59. In addition, MSCs have been shown to modulate the adaptive immune system as well, mainly through suppression of T cell proliferation 60. It is thought that these properties may ameliorate ischaemic cardiac damage especially during the initial immune response to injury.

Mesenchymal stem cells have been isolated from many different tissue types including bone marrow, adipose tissue, lung tissue, umbilical cord blood and peripheral blood, but are most easily harvested from the bone marrow and adipose tissue. In particular, adipose-derived mesenchymal stem cells (ADCs) have the attractive feature of being easily harvested and isolated from an allogeneic source through liposuction with a high yield. Thus, most pre-clinical and clinical studies have focused on delivery of MSCs isolated from these two sources. Large-animal studies reported the ability of MSCs to decrease infarct size and improve ventricular function 61,62. These studies used multiple delivery methods including intravenous injection, intracoronary infusion, catheter-based intramyocardial injection and direct surgical myocardial injection 49. As with other cell types studied for cardiac repair, the exact mechanisms for the improvement in heart function are unclear, but are likely related to possible anti-inflammatory effects as well as paracrine signalling to recruit endogenous stem cells and promote healing by minimizing fibrosis.

Based on the promising initial pre-clinical results, multiple clinical trials have evaluated the use of bone marrow and ADCs both in acute cardiac ischaemia as well as ischaemic cardiomyopathy. Studies involving intravenous 63 intracoronary infusion 64 and intramyocardial injection 65 of bone marrow derived MSCs as well as intracoronary infusion of ADCs (APOLLO) 66 have demonstrated safety of autologous and allogeneic cells in acute and subacute MI, with modest improvement in LV ejection fraction. Early clinical trials using MSCs in ischaemic cardiomyopathy, most notably TAC-HFT (Transendocardial Autologous Mesenchymal Stem Cells and Mononuclear Bone Marrow Cells in Ischemic Heart Failure Trial) 67 comparing MSCs with BM mononuclear cells and POSEIDON 68 comparing allogeneic with autologous MSCs appear to confirm the safety of this cell type, although determination of clinical efficacy will necessitate larger trials. The PROMETHEUS study 69 utilizing autologous MSCs in patients with chronic ischaemic cardiomyopathy undergoing coronary artery bypass grafting (CABG) points to efficacy based on improvement in ventricular contractile function and decrease in scar size. Two clinical studies looking at safety and efficacy of adipose-derived MSCs have recently been completed for both acute ischaemia 195 and chronic ischaemic cardiomyopathy 196, and the results of these studies will delineate the potential regenerative efficacy of this particular cell type in cardiac repair.

### Cardiac progenitor cells

Until recently, it was thought that the heart was a fully differentiated organ without the capacity for regeneration. Multiple groups 9, including ours 10, have since found that post-natal generation of new cardiomyocytes does indeed occur, albeit at a very low rate. Many types of putative ‘cardiac progenitor cells’ (CPCs) have been reported, with the shared definition that they are clonal multi-potent cells capable of self-renewal and differentiation into the three major cardiac cell types. The most clinically relevant, of these types for cell therapy have been the c-kit^+^ cell 70 and the cardiosphere-derived cell (CDC) 71, while Sca-1^+^ cells 72, Isl-1^+^ cells 73,74, SSEA-1^+^ cells 75,76, side-population cells 77 and telocytes 78,79 have also been the subject of research interest.

Cardiac progenitor cells with the ability to differentiate into cardiomyocytes, endothelial cells and smooth muscle cells were first reported in the rat heart by Beltrami *et al*. in 2003 70, and later in the human heart 80. These cells reportedly expressed the tyrosine kinase receptor c-kit (CD117), a marker of stemness, lacked hematopoetic lineage markers, and were found to be multi-potent, clonal and self-renewing 81. Early studies utilizing human c-kit^+^ CPCs in an infarction model of immunodeficient mice reported successful engraftment, differentiation into the three major cardiac cell types, and improvement in cardiac function by echocardiography 80. In a porcine chronic infarct model, c-kit^+^ CPCs were first isolated and expanded from right atrial appendage resections, and then delivered *via* coronary artery infusion through a catheter 82. Results showed successful engraftment of delivered cells and improvement in LV function, setting the stage for translation into human clinical trials. The SCIPIO 83 trial utilized autologous c-kit^+^ CPCs harvested and expanded from the right atrial appendage at the time of CABG, with intracoronary infusion at a mean of 113 days after CABG. At 1 year after infusion, LV function by echocardiography was found to increase by 12.3% ± 2.1% compared to the control group, while the infarct size by magnetic resonance imaging (MRI) was found to decrease significantly.

Another CPC type under intense investigation has been the CDC 84. First isolated from mice and human biopsy samples in 2004 71 and later in dogs 85,86, these cells were expanded using spheroid culture technique. These cells were then found to form aggregates of a heterogenous cell population that expressed stem cell markers such as c-kit, Sca-1 and CD34. Further characterization revealed multi-potentiality and clonogenicity of the cells, with cells at varying stages of differentiation (based on expression of cardiac lineage markers such as cardiac Troponin-I, atrial natriuretic peptide and CD31) depending on their location within the cell mass. The cells in the core were found to be mainly proliferating c-kit^+^ cells, with more differentiated cells as well as MSCs (characterized by expression of CD90 and CD105) towards the periphery, potentially indicating a role for MSCs in promoting CPC differentiation and renewal. The mediator for CDC-induced regeneration may be related to exosome delivery of miR-146a 87,88. More recently, it was found that THY-1 (Thymocyte antigen 1) (CD90) receptor expression could also be used to delineate CDCs with divergent cardiac differentiation potential into either mesenchymal/myofibroblast cells or cardiomyocytes 89. Initial pre-clinical studies involving injection of CDCs in an immunodeficient murine infarction model showed improvement in echocardiographic cardiac function 90. This led to a porcine study 91 using intracoronary delivery of CDCs which demonstrated reduction in relative infarct size by MRI. Soon thereafter, the initial human clinical trial (CADUCEUS) 92 studying autologous CDCs obtained through endomyocardial biopsy reported decreased scar size by MRI in patients receiving intracoronary infusion of CDCs after AMI.

The demonstration of clinical safety in both SCIPIO and CADUCEUS (along with suggestion of efficacy) has been encouraging for the field, but efficacy will have to be confirmed after longer time periods and through larger clinical trials involving sample sizes powered for such a determination. The ALLSTAR trial 191 investigating the delivery of allogeneic CDCs in patients with LV dysfunction after MI will shed more light on the future of this cell type as a therapeutic option.

### Pluripotent stem cells

Pluripotent stem cells have the ability to differentiate into all cell lineages, and hence offer novel treatment options for many intractable diseases including end-stage heart failure. Human embryonic stem cells (hESCs) have been investigated as a source of cells for cardiac repair through *ex vivo* differentiation into either cardiac ‘progenitors’ 76 or into mature cardiomyocytes 93. However, limitations include the inability to isolate pure tissue-specific progenitors capable of robust engraftment and regeneration, potential risk of teratoma formation from residual PSCs in the transplanted cells 94, and ethical concerns with their generation.^95^ In addition, it is uncertain that hESCs can functionally engraft and electromechanically couple into the surrounding myocardium. These concerns have limited the clinical translation of hESCs for cardiac therapy.

The report by Yamanaka in 2006 96 that terminally differentiated murine fibroblasts could be ‘reprogrammed’ to a primitive embryonic stem cell-like state through introduction of four specific transcription factors (Oct3/4, Sox2, c-Myc and Klf4) brought new hope to cardiac regenerative medicine. These cells, called induced PSCs (iPSCs), may bypass the ethical concerns associated with ESCs, and serve as a potentially unlimited source of cells for transplantation. While murine studies reported engraftment of iPSCs into infarcted myocardium 97, concerns for tumourgenicity have greatly limited further investigation using direct transplantation of iPSCs.

The most promising application of PSCs in cardiac regenerative medicine has been their use as a cell source for derivation of adult cardiomyocytes for transplantation. While early protocols for differentiating ESCs into cardiomyocytes generated less than 1% yields 93, more recent differentiation protocols have achieved yields of up to 70% 98. Further enrichment for ESC-derived cardiomyocytes can be accomplished through use of a cardiac-specific promoter for expression of a fluorescent protein 99, sorting for cell surface markers 100–102 or sorting *via* Raman spectroscopy 103. Our group has reported on hESC-derived ROR2(+)/CD13(+)/KDR(+)/PDGFRα(+) cells that give rise to cardiomyocytes 104 as well as endothelial cells and vascular smooth muscle cells. To date, ESCs, iPSCs 105 and even parthenogenetic PSCs 106 have been successfully differentiated into cardiomyocytes. Investigation into the electrical-mechanical properties of derived cardiomyocytes have found them to exhibit significant automaticity with immature action potential 107 and contractile 108 properties, highlighting the need for further development of differentiation conditions capable of producing cardiomyocytes of more mature phenotype compatible with native myocardium.

*In vivo* studies utilizing PSC-derived cardiomyocytes have been promising, with early rodent studies in acute 93 and chronic 109 infract models demonstrating improvement in ventricular contractile function. More recently, hESC-derived cardiomyocytes have been shown in a primate model of ischaemia-reperfusion injury to engraft into infarcted host tissue, ‘remuscularize’ the infarct region, and electromechanically couple to surrounding host cardiomyocytes 110. However, the presence of arrhythmias were reported in all animals receiving cell therapy, highlighting the potential problem with the arrhythmogenicity of transplanted cell Whether these cells are inherently arrhythmogenic or serve as a nidus to induce arrhythmias is still not entirely clear. Future translation of this approach will require further understanding to eliminate the arrhythmogenicity inherent in transplanted cardiomyocytes before human clinical studies can be initiated.

## Endogenous cell therapy

Cell therapy relying on exogenous delivery of cells has provided great promise for the treatment of cardiovascular diseases. However, issues such as low cell survival, poor engraftment and limited functional maturation have emphasized the need to develop novel therapeutic alternatives. Regeneration of cardiac tissue through use of endogenous cardiac cells, as with direct reprogramming of resident cardiac fibroblasts or stimulation of native cardiomyocyte proliferation, can potentially sidestep the inherent limitations of exogenous cell delivery.

### Direct reprogramming of endogenous cells

Shortly after Yamanaka’s report of reprogramming of somatic cells to iPSCs 96, the ability of these cells to differentiate into functional cardiomyocytes was readily demonstrated 105. However, as with ESCs, the utilization of iPSC-derived cardiomyocytes raised a number of concerns such as potential differentiation towards alternative cell fates and teratoma formation once introduced to the heart. Direct reprogramming of fibroblasts to cardiomyocytes bypassing the pluripotent state was proposed as a method overcoming such hurdles 111,112. The abundance of fibroblasts in the heart 113 as well as their role following injury highlight the therapeutic potential of this approach. Direct conversion of fibroblasts into cardiomyocytes was first reported by Ieda *et al*. 112. The authors showed that the combination of three transcription factors, GATA4, MEF2C and TBX5 (referred to as GMT) was able to convert mouse dermal and cardiac fibroblasts into cardiomyocyte-like cells, termed induced cardiomyocytes (iCMs). iCMs exhibited a gene expression profile similar to native cardiomyocytes while the fibroblast gene program was silenced, and a small fraction was able to spontaneously contract. However, the efficiency of the conversion was very low and the majority of iCMs was only partially reprogrammed. Similarly, Protze *et al*. demonstrated time-dependent conversion of mouse embryonic fibroblasts into cardiomyocyte-like cells through lentiviral expression of myocardin, MEF2C and TBX5 114, while Song *et al*. reported the requirement of four factors GATA4, HAND2, MEF2C and TBX5 (GHMT) 115. Although in the setting of the experiments by Ieda *et al*. 112, miRNAs were not required for reprogramming, Jayawardena *et al*. demonstrated that a panel of four miRNAs (miR-1, miR-133, miR-208 and miR-499) and a JAK inhibitor were sufficient for direct conversion of mouse fibroblasts into cells with characteristics of cardiomyocytes 116. Muraoka *et al*. demonstrated that addition of miR-133 to the GMT combination resulted in a sevenfold increase in the generation of beating cardiomyocytes 117. More recently, Addis *et al*. utilized a reporter system carrying the calcium indicator GCaMP under a Troponin-T promoter, and found that the combination of Hand2, Nkx2.5, Gata4, Mef2c and Tbx5 (HNGMT) was the most efficient in reprogramming of embryonic and adult mouse fibroblasts into functional cardiomyocytes 118. It was then shown that small molecule inhibition of TGF-β using SB432542 in combination with HNGMT further increased reprogramming efficiency 119. Besides the specific combination of factors, the stoichiometric expression of GMT factors significantly affects the efficiency of reprogramming and the quality of the iCMs 120. In an effort to develop clinically applicable strategies for direct reprogramming, Wang *et al*. identified a cocktail of small molecules that was sufficient to reprogram mouse fibroblasts to ventricular-like cardiomyocytes in the presence of only one transcription factor, Oct4 121.

However, the need for the development of regenerative strategies that do not require cell transplantation, as well as the low efficiency of direct reprogramming *in vitro* moved the field towards *in vivo* conversion of fibroblasts to cardiomyocyte-like cells 115,116,122,123. Retrovirus-mediated intramyocardial delivery of the GMT 122 or GHMT 115 combinations of transcription factors following MI resulted in successful direct reprogramming of fibroblasts into cardiomyocytes. The fraction of iCMs exhibiting characteristics of endogenous cardiomyocytes was significantly increased in the *in vivo* setting compared to *in vitro* reprogramming. Importantly the authors reported a decrease in infarct size and improvement in heart function 115,122. More recently, it was found that lentiviral-mediated administration of miR-1, miR-133, miR-208 and miR-499 into infarcted mouse hearts resulted in direct reprogramming of resident fibroblasts into cells with cardiomyocyte morphology and function, resulting in decreased infarct size and improved cardiac function 124.

Consistent with the findings in mice, recent studies have demonstrated the conversion of human fibroblasts to cells with cardiomyocyte characteristics 125–127. Although human cells have been proven to be more challenging, various combinations of transcription factors and miRNAs (GATA4, HAND2, TBX5, myocardin and the miRNAs miR-1 and miR-133 125, GMT, together with Myocardin, ZFPM2 (Zinc finger protein multitype 2) and TGF-β 126, and GMT in addition to Mesp1 and Myocd) 127 have successfully produced human cardiomyocyte-like cells. Further studies in larger animals are required to explore their *in vivo* reprogramming potential.

### Activation of endogenous cardiomyocytes

Mammalian cardiomyocytes have long been considered as post-mitotic, terminally differentiated cells unable to re-enter the cell cycle. A number of studies have challenged this dogma, providing evidence of cardiomyocyte division in the adult heart 7,9. However, although the neonatal heart exhibits a robust regenerative capacity following injury 128,129, in adults, the rate of cardiomyocyte proliferation is low and inadequate to replenish the lost tissue. In an effort to ‘re-activate’ mature cardiomyocytes, a number of studies have suggested a variety of molecules ranging from growth and transcription factors, to cell cycle genes, to miRNAs, as potential therapeutic means to promote endogenous cardiomyocyte proliferation 130.

Administration of periostin, an extracellular matrix (ECM) protein produced by fibroblasts, has been shown to improve cardiac function and decrease infarct size following MI 131–133. The beneficial effects of periostin have been attributed to increased cardiomyocyte DNA synthesis, mitosis and cytokinesis as well as increased angiogenesis 131–133. However, the use of periostin as a therapeutic agent remains controversial 133,134, and further investigation is required.

Neuregulin is another protein that has exhibited strong therapeutic potential. This growth factor has been shown to promote cardiomyocyte cell cycle re-entry and cytokinesis in a mouse infarction model in addition to a pro-angiogenic and anti-apoptotic function 135. Neuregulin treatment resulted in reduced scar size, ameliorated heart function and decreased hypertrophy 136. More recently, Polizzotti *et al*. reported the existence of a ‘therapeutic window’, confined to the first postnatal days in mice and the first 6 months in humans, during which neuregulin treatment has remarkably higher efficiency in promoting cardiomyocyte regeneration 137. Similarly, expression of the neuregulin co-receptor ERBB2 (human epidermal growth factor receptor 2) was shown to be sufficient for cardiomyocyte proliferation and tissue regeneration following injury 138. Phase II clinical trials examining neuregulin administration as a therapeutic alternative for heart failure, have produced very promising results 139.

Insulin growth factor 1 (IGF1) and fibroblast growth factor 1 (FGF1) have also been proposed to promote cardiomyocyte proliferation 140–142. Mice over-expressing IGF1 specifically in cardiomyocytes exhibited larger hearts as a result of cardiomyocyte hyperplasia 140, while in a recent report it was suggested that activation of the IGF1/Akt pathway coincides with a ‘proliferative burst’ of cardiomyocytes in preadolescent mice 141. Likewise, Engel *et al*. demonstrated that a combination of FGF1 and a p38 inhibitor improved heart function and cardiomyocyte cycling in rats following MI 142. Growth factor pathways such as IGF, Hedgehog and TGF-β were also identified through an *in vivo* screening of cardiomyocyte proliferation modifiers 143.

In addition to the administration of exogenous proteins, alteration in the expression of transcription factors 144 as well as cell cycle genes 145,146 represents an alternative therapeutic option. Namely, cardiomyocyte-specific deletion of a member of the TALE family of transcription factors (including Meis1) extended the proliferative window of postnatal cardiomyocytes from 7 to 14 days, while its overexpression reduced cardiomyocyte proliferation and decreased neonatal cardiac regeneration 144. In the same context, Cheng *et al*. showed that constitutive myocardial expression of Cyclin A2 in mice resulted in enhanced cardiac function explained by cardiomyocyte cell cycle re-entry and increased regeneration 145. More recently, adenovirus mediated delivery of Cyclin A2 in the peri-infarct area of pig hearts produced similar results 146.

Finally, several miRNAs involved in the regulation of cardiomyocyte proliferation have been proposed as potential therapeutic candidates 129,147. Porrello *et al*. elegantly demonstrated that inhibition of the miR-15 family results in cardiomyocyte proliferation and improved cardiac function following infarction in adult mice 129. High-throughput screening of human miRNAs revealed forty miRNAs regulating cardiomyocyte DNA synthesis and cytokinesis *in vitro* while two of these (has-miR-590 and has-miR-199a) promoted cardiac regeneration and restored cardiac function in a mouse infarction model 147. Similarly, the microRNA cluster miR-302-367 was shown to activate cardiomyocyte cell cycle re-entry and proliferation as well as decrease scar formation following MI in mice, partly because of inhibition of the organ size control signalling pathway Hippo 148. These recent data are in agreement with a number of studies which indicated that inactivation of the Hippo pathway promotes cardiomyocyte proliferation and cytokinesis after injury in both neonatal and adult mice 149, and that activation of the Hippo pathway effector protein Yap stimulates cardiomyocyte regeneration and improves cardiac function after injury in mice 150–152.

Development of novel therapies based on the delivery of molecules that are able to stimulate the endogenous cardiac cells to undergo proliferation offers significant advantages. Myocardial regeneration strategies, whether they involve fibroblast reprogramming or cardiomyocyte cell cycle re-entry would circumvent issues associated with more invasive cell-based therapies such as cell survival, engraftment and electromechanical coupling with resident cells.

## Future directions

As described in previous sections, the transplantation of several cell types has been shown in multiple pre-clinical and clinical studies to be a safe technique for potentially improving cardiac function, although evidence for true cardiac regeneration through successful engraftment of exogenous cells has been limited. This perhaps should not be surprising given the complexity of cardiac tissue. Multiple factors likely play a role in early cell death after intramyocardial delivery of exogenous cells, including the absence of necessary survival factors in the transplanted cells 153, loss of physiological signalling through interactions with the ECM 154, limited vascular supply in the local microenvironment 155, an inflammatory milieu in the aftermath of cell delivery 156 and inability to electromechanically couple with the host cardiomyocytes 16. Strategies to improve cell retention and proliferation have thus focused on improving the immediate microenvironment into which the cells are delivered. One approach has investigated simultaneous delivery of pro-survival growth factors 93,131,157, of which the ideal combination has yet to be identified. Another technique being studied is the ‘pre-conditioning’ of cells into a pro-survival state through exposure to ischaemia, cytokines or heat shock 156.

Of particular interest are two approaches that seek to more closely recapitulate the particular microenvironment of a cardiac stem cell ‘niche’ to improve cell engraftment and survival. First, the co-delivery of two (or more) different types of cells takes advantage of potential synergistic and complimentary interactions between different cell populations. Second, bioengineering approaches such as the seeding and delivery of tissue engineered scaffolds could potentially enhance survival of delivered cells by providing the microstructural framework and extracellular cues necessary for cell viability.

### Combination cell therapy

The ‘niche’ model of adult stem cell self-renewal and differentiation, originally developed by Schofield in 1978 158, describes a local microenvironment in which tissue (including cardiac tissue) is generated, maintained and repaired by stem cells under the regulation of a complex interaction between the stem cells and surrounding niche support cells, soluble signalling molecules, and interactions with the ECM 159,160. As a large percentage of heart volume is comprised of interstitial tissue, it is thought that cardiac niches within the interstitial compartments of the myocardium and epicardium are responsible for potential cardiac regeneration. In particular, MSCs have been found to be involved in regulation of the cardiac as well as HSC niches 161. Combination cell therapy builds upon this framework by co-delivering stromal support cells with stem cells to enhance cell survival and engraftment into the surrounding tissue. An early demonstration of this concept involved co-transplantation of satellite cells and MSCs in a murine model of Chagas cardiomyopathy, a combination which was found to improve cardiac function compared to control 162. In another study, the combination of EPCs and MSCs was found to synergistically form functional vascular networks in Matrigel that remained patent at 4 weeks *in vivo* 163.

Another stromal support cell called the telocyte has been found to closely relate to CPCs at the level of the stem cell niche 164,165 by directing progenitor cell differentiation *via* microRNA vesicular transfer 166,167, making it another potential cell type for combination therapy. Separate from cardiac fibroblasts 168, these cells have been studied to improve cardiac function in rat models of MI 169,170.

More recently, it has been found that MSCs induce proliferation and differentiation of c-kit^+^ CPCs *via* interactions through connexin-43 gap junctions 161. Based on this understanding, a combination approach using both MSCs and c-kit^+^ CPCs was found to be synergistic in reducing scar size and improving cardiac function in a porcine model of MI when compared to either cell type alone 171. A clinical trial to further evaluate this approach (AIRMID) is currently in the planning stage, and may further advance this field.

Another potential approach combines c-kit^+^ CPCs with pericytes, a support cell thought to play an important role in vascular growth and angiogenesis through paracrine mediators 172. An early-stage murine study demonstrated improved cardiac contractility by echocardiography as well as improved vascular proliferation and arteriogenesis 173, but further study will be required before such an approach can be translated to clinical trials.

### Bioengineering approaches

Normal functioning myocardium relies on a complex and dynamic interaction between multiple cell types, the ECM, and soluble signalling factors. In particular, an adult CPC ‘niche’ is governed by diverse interactions between surface-bound integrins (such as α1β1, α2β1, α10β1, α11β1 integrins) 174 and the ECM proteins collagen, elastin, laminin and fibronectin 175,176. The low rates of cell survival and engraftment in exogenous cell therapy is thus likely related in part to the dearth of these important physiological cues necessary for homeostasis immediately during and after delivery 177.

The ideal biomaterial complement to cell therapy should provide a proper three-dimensional structure with appropriate biological, bioelectrical, biomechanical and biochemical features specific for the cell type 178,179. Much attention has been focused on the incorporation of signalling molecules to influence cell biology. Strategies to date have ranged from co-delivery of ECM components such as collagen 180, Matrigel 181, fibrinogen 182, and de-cellularized ECM 183,184, to non-ECM biological materials such as chitosan 185, to *in vitro* construction of seeded tissue-engineered scaffolds transplanted as cardiac patches 186. Synthetic materials can be designed for specific properties; poly(lactic-*co*-glycolic acid) microcarriers can release growth factors in concert with co-delivered cells 187, and self-assembling peptide nanofibers can be co-delivered with cells to improve cell retention, direct differentiation and deliver protein 188–190. However, further study regarding materials biocompatibility and biodegradation will be required prior to further clinical translation of this technology. Future efforts to develop resorbable, electrically conductive and biologically active materials with minimal modulus mismatch and adequate immunomodulatory properties would significantly advance this field. In addition, advances in tools and technologies to promote targeted delivery of progenitor cells to ischaemic and infarcted tissues as well as improvements in non-invasive cell tracking will reveal new insights on cell survival and integration.

## Conclusions

Cell-based therapy for amelioration and regeneration of cardiac tissue has been widely studied as a novel approach for the treatment of ischaemic heart disease. Multiple cell types have been intensely characterized and investigated as potential candidates for exogenous delivery. Initial studies using skeletal myoblasts, while encouraging in animal models, highlighted the inherent arrhythmogenic potential of exogenous cells that do not integrate electrically with the surrounding myocardium. Bone marrow cells, in both unselected and purified forms, have been under wide clinical investigation despite inconsistent outcomes. The unique immunomodulatory properties of MSCs may make them excellent candidates for combined therapy with another cell type. Pluripotent stem cells have emerged as an almost limitless source for derivation of differentiated cardiomyocytes with the potential to physiologically integrate with host myocardium both electrically and mechanically. Perhaps the cell types with some potential for cardiac repair have been the c-kit+ and cardiosphere-derived CPCs, although independent large clinical trials are needed to confirm the preliminary results.

**Figure 2:**
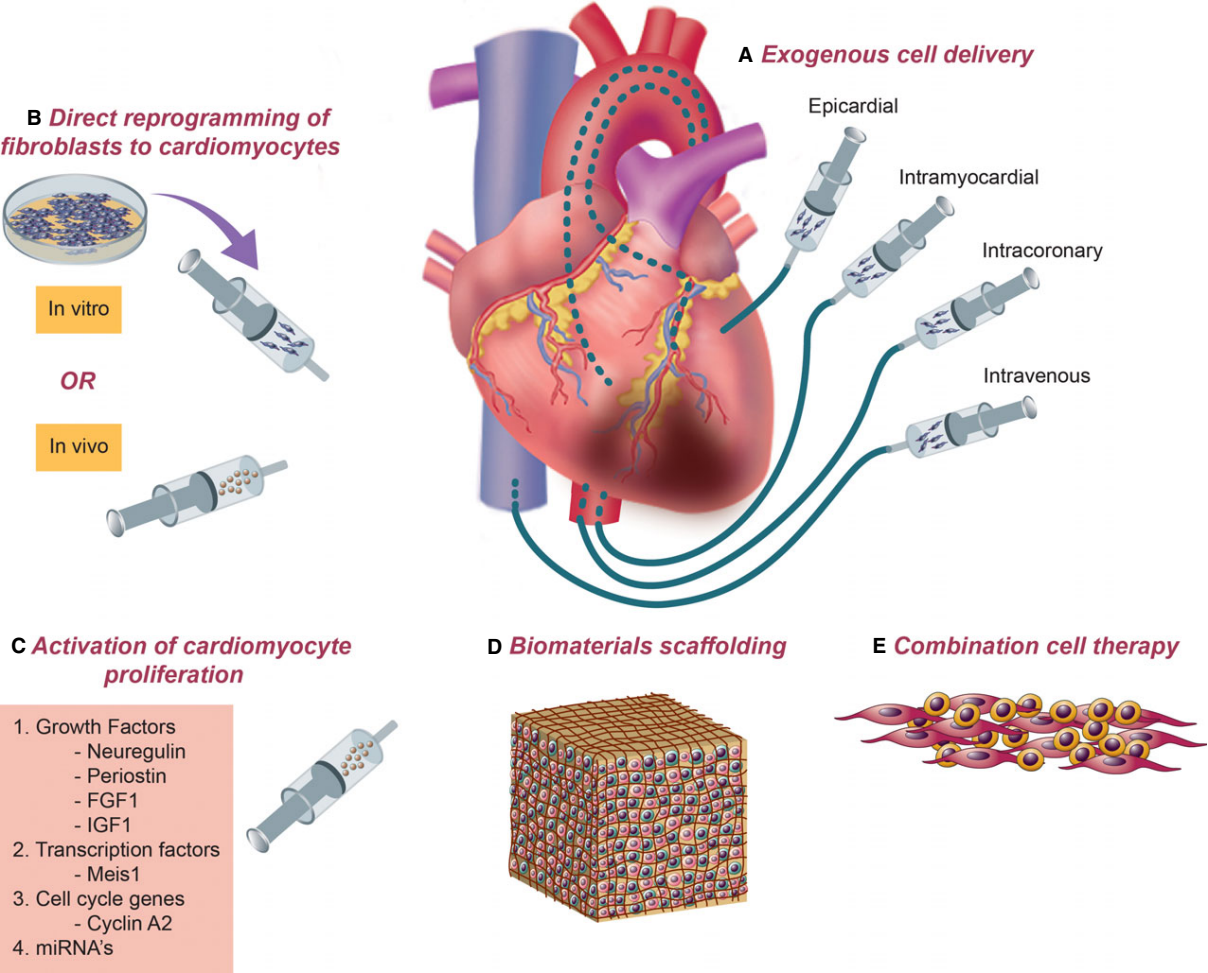
A combined approach for amelioration of injury and rejuvenation of cardiac tissue. Successful cardiac regeneration will likely necessitate a combination of therapeutic approaches. (A) Delivery of exogenous cells has been demonstrated *via* epicardial, intramyocardial (endocardial), intracoronary and intravenous routes. (B) Fibroblasts directly reprogrammed into cardiomyocytes either *in vitro* or *in vivo* can potentially serve as an abundant source of cells for cardiac regeneration. (C) Stimulation of native cardiomyocyte proliferation may be possible using a number of protein- and nucleic acid- based factors. Delivery of multiple cell types (E) along with delivery of biomaterials-based scaffolding (D) may be necessary for optimal cell engraftment and tissue regeneration.

Despite these advances, significant obstacles remain in the field; low cell survival, poor engraftment and limited functional maturation (of progenitor cells) have blunted potential therapeutic benefit. While from a putative standpoint one would expect cell therapy to exert its beneficial effect by repopulating the damaged myocardium by the exogenous cells, others have argued that the delivery of exogenous cells may lead to recruitment of intrinsic cells capable of regenerating the damaged muscle; hence the loss of transplanted cells after a short time does not preclude the promise of stem cell therapy. Cell therapy strategies involving direct reprogramming of endogenous cardiac fibroblasts into cardiomyocytes and stimulation of endogenous cardiomyocyte expansion through growth and transcription factor delivery have the potential to sidestep the inherent limitations of exogenous cell delivery. Ultimate success with cardiac cell therapy will likely necessitate a combined strategy involving exogenous delivery of multiple complementary cell types, soluble factors for enhanced cell survival, concurrent stimulation of endogenous cardiomyocyte regeneration, recruitment and transdifferentiation of endogenous cardiac fibroblasts into cardiomyocytes through direct reprogramming, and the use of biomaterial scaffolds to provide structural support and biochemical cues during delivery (Figure2).

## References

[b1] Go AS, Mozaffarian D, Roger VL (2014). Executive summary: heart disease and stroke statistics–2014 update: a report from the American Heart Association. Circulation.

[b2] Chen J, Normand SL, Wang Y (2011). National and regional trends in heart failure hospitalization and mortality rates for Medicare beneficiaries, 1998-2008. JAMA.

[b3] Yancy CW, Jessup M, Bozkurt B (2013). ACCF/AHA guideline for the management of heart failure: a report of the American College of Cardiology Foundation/American Heart Association Task Force on Practice Guidelines. J Am Coll Cardiol.

[b4] Murry CE, Reinecke H, Pabon LM (2006). Regeneration gaps: observations on stem cells and cardiac repair. J Am Coll Cardiol.

[b5] Duncan AW, Dorrell C, Grompe M (2009). Stem cells and liver regeneration. Gastroenterology.

[b6] Oberpriller JO, Oberpriller JC (1974). Response of the adult newt ventricle to injury. J Exp Zool.

[b7] Bergmann O, Bhardwaj RD, Bernard S (2009). Evidence for cardiomyocyte renewal in humans. Science.

[b8] Orlic D, Kajstura J, Chimenti S (2001). Bone marrow cells regenerate infarcted myocardium. Nature.

[b9] Senyo SE, Steinhauser ML, Pizzimenti CL (2013). Mammalian heart renewal by pre-existing cardiomyocytes. Nature.

[b10] Ali SR, Hippenmeyer S, Saadat LV (2014). Existing cardiomyocytes generate cardiomyocytes at a low rate after birth in mice. Proc Natl Acad Sci USA.

[b11] Brack AS, Rando TA (2012). Tissue-specific stem cells: lessons from the skeletal muscle satellite cell. Cell Stem Cell.

[b12] Taylor DA, Atkins BZ, Hungspreugs P (1998). Regenerating functional myocardium: improved performance after skeletal myoblast transplantation. Nat Med.

[b13] Menasche P (2008). Skeletal myoblasts for cardiac repair: Act II?. J Am Coll Cardiol.

[b14] Menasche P, Alfieri O, Janssens S (2008). The Myoblast Autologous Grafting in Ischemic Cardiomyopathy (MAGIC) trial: first randomized placebo-controlled study of myoblast transplantation. Circulation.

[b15] Povsic TJ, O’Connor CM, Henry T (2011). A double-blind, randomized, controlled, multicenter study to assess the safety and cardiovascular effects of skeletal myoblast implantation by catheter delivery in patients with chronic heart failure after myocardial infarction. Am Heart J.

[b16] Araya R, Eckardt D, Maxeiner S (2005). Expression of connexins during differentiation and regeneration of skeletal muscle: functional relevance of connexin43. J Cell Sci.

[b17] Gepstein L, Ding C, Rahmutula D (2010). *In vivo* assessment of the electrophysiological integration and arrhythmogenic risk of myocardial cell transplantation strategies. Stem Cells.

[b18] Reinecke H, Poppa V, Murry CE (2002). Skeletal muscle stem cells do not transdifferentiate into cardiomyocytes after cardiac grafting. J Mol Cell Cardiol.

[b19] Murry CE, Soonpaa MH, Reinecke H (2004). Haematopoietic stem cells do not transdifferentiate into cardiac myocytes in myocardial infarcts. Nature.

[b20] Balsam LB, Wagers AJ, Christensen JL (2004). Haematopoietic stem cells adopt mature haematopoietic fates in ischaemic myocardium. Nature.

[b21] Kocher AA, Schuster MD, Szabolcs MJ (2001). Neovascularization of ischemic myocardium by human bone-marrow-derived angioblasts prevents cardiomyocyte apoptosis, reduces remodeling and improves cardiac function. Nat Med.

[b22] Orlic D, Kajstura J, Chimenti S (2003). Bone marrow stem cells regenerate infarcted myocardium. Pediatr Transplant.

[b23] Dimmeler S, Burchfield J, Zeiher AM (2008). Cell-based therapy of myocardial infarction. Arterioscler Thromb Vasc Biol.

[b24] Martin-Rendon E, Brunskill SJ, Hyde CJ (2008). Autologous bone marrow stem cells to treat acute myocardial infarction: a systematic review. Eur Heart J.

[b25] Challen GA, Boles NC, Chambers SM (2010). Distinct hematopoietic stem cell subtypes are differentially regulated by TGF-beta1. Cell Stem Cell.

[b26] Asahara T, Murohara T, Sullivan A (1997). Isolation of putative progenitor endothelial cells for angiogenesis. Science.

[b27] Urbich C, Dimmeler S (2004). Endothelial progenitor cells: characterization and role in vascular biology. Circ Res.

[b28] Kawamoto A, Gwon HC, Iwaguro H (2001). Therapeutic potential of *ex vivo* expanded endothelial progenitor cells for myocardial ischemia. Circulation.

[b29] Kawamoto A, Tkebuchava T, Yamaguchi J (2003). Intramyocardial transplantation of autologous endothelial progenitor cells for therapeutic neovascularization of myocardial ischemia. Circulation.

[b30] Losordo DW, Henry TD, Davidson C (2011). Intramyocardial, autologous CD34^+^ cell therapy for refractory angina. Circ Res.

[b31] Schachinger V, Assmus B, Britten MB (2004). Transplantation of progenitor cells and regeneration enhancement in acute myocardial infarction: final one-year results of the TOPCARE-AMI Trial. J Am Coll Cardiol.

[b32] Assmus B, Honold J, Schachinger V (2006). Transcoronary transplantation of progenitor cells after myocardial infarction. N Engl J Med.

[b33] Povsic TJ, Junge C, Nada A (2013). A phase 3, randomized, double-blinded, active-controlled, unblinded standard of care study assessing the efficacy and safety of intramyocardial autologous CD34^+^ cell administration in patients with refractory angina: design of the RENEW study. Am Heart J.

[b34] Shantsila E, Watson T, Lip GY (2007). Endothelial progenitor cells in cardiovascular disorders. J Am Coll Cardiol.

[b35] Takano H, Ohtsuka M, Akazawa H (2003). Pleiotropic effects of cytokines on acute myocardial infarction: G-CSF as a novel therapy for acute myocardial infarction. Curr Pharm Des.

[b36] Takahashi T, Kalka C, Masuda H (1999). Ischemia- and cytokine-induced mobilization of bone marrow-derived endothelial progenitor cells for neovascularization. Nat Med.

[b37] Yano T, Miura T, Whittaker P (2006). Macrophage colony-stimulating factor treatment after myocardial infarction attenuates left ventricular dysfunction by accelerating infarct repair. J Am Coll Cardiol.

[b38] Lee M, Aoki M, Kondo T (2005). Therapeutic angiogenesis with intramuscular injection of low-dose recombinant granulocyte-colony stimulating factor. Arterioscler Thromb Vasc Biol.

[b39] Misao Y, Takemura G, Arai M (2006). Importance of recruitment of bone marrow-derived CXCR4^+^ cells in post-infarct cardiac repair mediated by G-CSF. Cardiovasc Res.

[b40] Orlic D, Kajstura J, Chimenti S (2001). Mobilized bone marrow cells repair the infarcted heart, improving function and survival. Proc Natl Acad Sci USA.

[b41] Deindl E, Zaruba MM, Brunner S (2006). G-CSF administration after myocardial infarction in mice attenuates late ischemic cardiomyopathy by enhanced arteriogenesis. FASEB J.

[b42] Takano H, Qin Y, Hasegawa H (2006). Effects of G-CSF on left ventricular remodeling and heart failure after acute myocardial infarction. J Mol Med.

[b43] Zohlnhofer D, Ott I, Mehilli J (2006). Stem cell mobilization by granulocyte colony-stimulating factor in patients with acute myocardial infarction: a randomized controlled trial. JAMA.

[b44] Ripa RS, Jorgensen E, Wang Y (2006). Stem cell mobilization induced by subcutaneous granulocyte-colony stimulating factor to improve cardiac regeneration after acute ST-elevation myocardial infarction: result of the double-blind, randomized, placebo-controlled stem cells in myocardial infarction (STEMMI) trial. Circulation.

[b45] Engelmann MG, Theiss HD, Hennig-Theiss C (2006). Autologous bone marrow stem cell mobilization induced by granulocyte colony-stimulating factor after subacute ST-segment elevation myocardial infarction undergoing late revascularization: final results from the G-CSF-STEMI (Granulocyte Colony-Stimulating Factor ST-Segment Elevation Myocardial Infarction) trial. J Am Coll Cardiol.

[b46] Ellis SG, Penn MS, Bolwell B (2006). Granulocyte colony stimulating factor in patients with large acute myocardial infarction: results of a pilot dose-escalation randomized trial. Am Heart J.

[b47] Simari RD, Pepine CJ, Traverse JH (2014). Bone marrow mononuclear cell therapy for acute myocardial infarction: a perspective from the cardiovascular cell therapy research network. Circ Res.

[b48] Friedenstein AJ, Chailakhjan RK, Lalykina KS (1970). The development of fibroblast colonies in monolayer cultures of guinea-pig bone marrow and spleen cells. Cell Tissue Kinet.

[b49] Williams AR, Hare JM (2011). Mesenchymal stem cells: biology, pathophysiology, translational findings, and therapeutic implications for cardiac disease. Circ Res.

[b50] Kim PJ, Mahmoudi M, Ge X (2015). Direct evaluation of myocardial viability and stem cell engraftment demonstrates salvage of the injured myocardium. Circ Res.

[b51] Toma C, Pittenger MF, Cahill KS (2002). Human mesenchymal stem cells differentiate to a cardiomyocyte phenotype in the adult murine heart. Circulation.

[b52] Xu WR, Zhang XR, Qian H (2004). Mesenchymal stem cells from adult human bone marrow differentiate into a cardiomyocyte phenotype *in vitro*. Exp Biol Med.

[b53] Koninckx R, Hensen K, Daniels A (2009). Human bone marrow stem cells co-cultured with neonatal rat cardiomyocytes display limited cardiomyogenic plasticity. Cytotherapy.

[b54] Acquistapace A, Bru T, Lesault PF (2011). Human mesenchymal stem cells reprogram adult cardiomyocytes toward a progenitor-like state through partial cell fusion and mitochondria transfer. Stem Cells.

[b55] Karantalis V, Hare JM (2015). Use of mesenchymal stem cells for therapy of cardiac disease. Circ Res.

[b56] Ema H, Suda T (2012). Two anatomically distinct niches regulate stem cell activity. Blood.

[b57] Dominici M, Le Blanc K, Mueller I (2006). Minimal criteria for defining multipotent mesenchymal stromal cells. The International Society for Cellular Therapy position statement. Cytotherapy.

[b58] Raffaghello L, Bianchi G, Bertolotto M (2008). Human mesenchymal stem cells inhibit neutrophil apoptosis: a model for neutrophil preservation in the bone marrow niche. Stem Cells.

[b59] Aggarwal S, Pittenger MF (2005). Human mesenchymal stem cells modulate allogeneic immune cell responses. Blood.

[b60] Di Nicola M, Carlo-Stella C, Magni M (2002). Human bone marrow stromal cells suppress T-lymphocyte proliferation induced by cellular or nonspecific mitogenic stimuli. Blood.

[b61] Quevedo HC, Hatzistergos KE, Oskouei BN (2009). Allogeneic mesenchymal stem cells restore cardiac function in chronic ischemic cardiomyopathy *via* trilineage differentiating capacity. Proc Natl Acad Sci USA.

[b62] Schuleri KH, Feigenbaum GS, Centola M (2009). Autologous mesenchymal stem cells produce reverse remodelling in chronic ischaemic cardiomyopathy. Eur Heart J.

[b63] Hare JM, Traverse JH, Henry TD (2009). A randomized, double-blind, placebo-controlled, dose-escalation study of intravenous adult human mesenchymal stem cells (prochymal) after acute myocardial infarction. J Am Coll Cardiol.

[b64] Chen SL, Fang WW, Ye F (2004). Effect on left ventricular function of intracoronary transplantation of autologous bone marrow mesenchymal stem cell in patients with acute myocardial infarction. Am J Cardiol.

[b65] Williams AR, Trachtenberg B, Velazquez DL (2011). Intramyocardial stem cell injection in patients with ischemic cardiomyopathy: functional recovery and reverse remodeling. Circ Res.

[b66] Houtgraaf JH, den Dekker WK, van Dalen BM (2012). First experience in humans using adipose tissue-derived regenerative cells in the treatment of patients with ST-segment elevation myocardial infarction. J Am Coll Cardiol.

[b67] Heldman AW, DiFede DL, Fishman JE (2014). Transendocardial mesenchymal stem cells and mononuclear bone marrow cells for ischemic cardiomyopathy: the TAC-HFT randomized trial. JAMA.

[b68] Hare JM, Fishman JE, Gerstenblith G (2012). Comparison of allogeneic *vs* autologous bone marrow-derived mesenchymal stem cells delivered by transendocardial injection in patients with ischemic cardiomyopathy: the POSEIDON randomized trial. JAMA.

[b69] Karantalis V, DiFede DL, Gerstenblith G (2014). Autologous mesenchymal stem cells produce concordant improvements in regional function, tissue perfusion, and fibrotic burden when administered to patients undergoing coronary artery bypass grafting: the Prospective Randomized Study of Mesenchymal Stem Cell Therapy in Patients Undergoing Cardiac Surgery (PROMETHEUS) trial. Circ Res.

[b70] Beltrami AP, Barlucchi L, Torella D (2003). Adult cardiac stem cells are multipotent and support myocardial regeneration. Cell.

[b71] Messina E, De Angelis L, Frati G (2004). Isolation and expansion of adult cardiac stem cells from human and murine heart. Circ Res.

[b72] Feng Y, Huang W, Meng W (2014). Heat shock improves Sca-1^+^ stem cell survival and directs ischemic cardiomyocytes toward a prosurvival phenotype *via* exosomal transfer: a critical role for HSF1/miR-34a/HSP70 pathway. Stem Cells.

[b73] Cai CL, Liang XQ, Shi YQ (2003). Isl1 identifies a cardiac progenitor population that proliferates prior to differentiation and contributes a majority of cells to the heart. Dev Cell.

[b74] Moretti A, Caron L, Nakano A (2006). Multipotent embryonic isl1^+^ progenitor cells lead to cardiac, smooth muscle, and endothelial cell diversification. Cell.

[b75] Ott HC, Matthiesen TS, Brechtken J (2007). The adult human heart as a source for stem cells: repair strategies with embryonic-like progenitor cells. Nat Clin Pract Cardiovasc Med.

[b76] Menasche P, Vanneaux V, Fabreguettes JR (2015). Towards a clinical use of human embryonic stem cell-derived cardiac progenitors: a translational experience. Eur Heart J.

[b77] Hierlihy AM, Seale P, Lobe CG (2002). The post-natal heart contains a myocardial stem cell population. FEBS Lett.

[b78] Cretoiu SM, Popescu LM (2014). Telocytes revisited. Biomol Concepts.

[b79] Tao L, Wang H, Wang X (2015). Cardiac telocytes. Curr Stem Cell Res Ther.

[b80] Bearzi C, Rota M, Hosoda T (2007). Human cardiac stem cells. Proc Natl Acad Sci USA.

[b81] Nigro P, Perrucci GL, Gowran A (2015). c-kit(+) cells: the tell-tale heart of cardiac regeneration?. Cell Mol Life Sci.

[b82] Bolli R, Tang XL, Sanganalmath SK (2013). Intracoronary delivery of autologous cardiac stem cells improves cardiac function in a porcine model of chronic ischemic cardiomyopathy. Circulation.

[b83] Bolli R, Chugh AR, D’Amario D (2011). Cardiac stem cells in patients with ischaemic cardiomyopathy (SCIPIO): initial results of a randomised phase 1 trial. Lancet.

[b84] Marban E (2014). Breakthroughs in cell therapy for heart disease: focus on cardiosphere-derived cells. Mayo Clin Proc.

[b85] Hensley MT, de Andrade J, Keene B (2015). Cardiac regenerative potential of cardiosphere-derived cells from adult dog hearts. J Cell Mol Med.

[b86] Yee K, Malliaras K, Kanazawa H (2014). Allogeneic cardiospheres delivered *via* percutaneous transendocardial injection increase viable myocardium, decrease scar size, and attenuate cardiac dilatation in porcine ischemic cardiomyopathy. PLoS ONE.

[b87] Ibrahim AG, Cheng K, Marban E (2014). Exosomes as critical agents of cardiac regeneration triggered by cell therapy. Stem Cell Reports.

[b88] Barile L, Gherghiceanu M, Popescu LM (2012). Ultrastructural evidence of exosome secretion by progenitor cells in adult mouse myocardium and adult human cardiospheres. J Biomed Biotechnol.

[b89] Gago-Lopez N, Awaji O, Zhang Y (2014). THY-1 receptor expression differentiates cardiosphere-derived cells with divergent cardiogenic differentiation potential. Stem Cell Reports.

[b90] Smith RR, Barile L, Cho HC (2007). Regenerative potential of cardiosphere-derived cells expanded from percutaneous endomyocardial biopsy specimens. Circulation.

[b91] Johnston PV, Sasano T, Mills K (2009). Engraftment, differentiation, and functional benefits of autologous cardiosphere-derived cells in porcine ischemic cardiomyopathy. Circulation.

[b92] Makkar RR, Smith RR, Cheng K (2012). Intracoronary cardiosphere-derived cells for heart regeneration after myocardial infarction (CADUCEUS): a prospective, randomised phase 1 trial. Lancet.

[b93] Laflamme MA, Chen KY, Naumova AV (2007). Cardiomyocytes derived from human embryonic stem cells in pro-survival factors enhance function of infarcted rat hearts. Nat Biotechnol.

[b94] Cao F, Lin S, Xie X (2006). *In vivo* visualization of embryonic stem cell survival, proliferation, and migration after cardiac delivery. Circulation.

[b95] Robertson JA (2001). Human embryonic stem cell research: ethical and legal issues. Nat Rev Genet.

[b96] Takahashi K, Yamanaka S (2006). Induction of pluripotent stem cells from mouse embryonic and adult fibroblast cultures by defined factors. Cell.

[b97] Nelson TJ, Martinez-Fernandez A, Yamada S (2009). Repair of acute myocardial infarction by human stemness factors induced pluripotent stem cells. Circulation.

[b98] Yang L, Soonpaa MH, Adler ED (2008). Human cardiovascular progenitor cells develop from a KDR^+^ embryonic-stem-cell-derived population. Nature.

[b99] Zandstra PW, Bauwens C, Yin T (2003). Scalable production of embryonic stem cell-derived cardiomyocytes. Tissue Eng.

[b100] Dubois NC, Craft AM, Sharma P (2011). SIRPA is a specific cell-surface marker for isolating cardiomyocytes derived from human pluripotent stem cells. Nat Biotechnol.

[b101] Uosaki H, Fukushima H, Takeuchi A (2011). Efficient and scalable purification of cardiomyocytes from human embryonic and induced pluripotent stem cells by VCAM1 surface expression. PLoS ONE.

[b102] Van Hoof D, Dormeyer W, Braam SR (2010). Identification of cell surface proteins for antibody-based selection of human embryonic stem cell-derived cardiomyocytes. J Proteome Res.

[b103] Pascut FC, Goh HT, George V (2011). Toward label-free Raman-activated cell sorting of cardiomyocytes derived from human embryonic stem cells. J Biomed Optics.

[b104] Ardehali R, Ali SR, Inlay MA (2013). Prospective isolation of human embryonic stem cell-derived cardiovascular progenitors that integrate into human fetal heart tissue. Proc Natl Acad Sci USA.

[b105] Zhang J, Wilson GF, Soerens AG (2009). Functional cardiomyocytes derived from human induced pluripotent stem cells. Circ Res.

[b106] Didie M, Christalla P, Rubart M (2013). Parthenogenetic stem cells for tissue-engineered heart repair. J Clin Invest.

[b107] Mummery C, Ward-van Oostwaard D, Doevendans P (2003). Differentiation of human embryonic stem cells to cardiomyocytes: role of coculture with visceral endoderm-like cells. Circulation.

[b108] Binah O, Dolnikov K, Sadan O (2007). Functional and developmental properties of human embryonic stem cells-derived cardiomyocytes. J Electrocardiol.

[b109] Fernandes S, Naumova AV, Zhu WZ (2010). Human embryonic stem cell-derived cardiomyocytes engraft but do not alter cardiac remodeling after chronic infarction in rats. J Mol Cell Cardiol.

[b110] Chong JJ, Yang X, Don CW (2014). Human embryonic-stem-cell-derived cardiomyocytes regenerate non-human primate hearts. Nature.

[b111] Vierbuchen T, Ostermeier A, Pang ZP (2010). Direct conversion of fibroblasts to functional neurons by defined factors. Nature.

[b112] Ieda M, Fu J-D, Delgado-Olguin P (2010). Direct reprogramming of fibroblasts into functional cardiomyocytes by defined factors. Cell.

[b113] Snider P, Hinton RB, Moreno-Rodriguez RA (2008). Periostin is required for maturation and extracellular matrix stabilization of noncardiomyocyte lineages of the heart. Circ Res.

[b114] Protze S, Khattak S, Poulet C (2012). A new approach to transcription factor screening for reprogramming of fibroblasts to cardiomyocyte-like cells. J Mol Cell Cardiol.

[b115] Song K, Nam Y-J, Luo X (2012). Heart repair by reprogramming non-myocytes with cardiac transcription factors. Nature.

[b116] Jayawardena TM, Egemnazarov B, Finch EA (2012). MicroRNA-mediated *in vitro* and *in vivo* direct reprogramming of cardiac fibroblasts to cardiomyocytes. Circ Res.

[b117] Muraoka N, Yamakawa H, Miyamoto K (2014). MiR-133 promotes cardiac reprogramming by directly repressing Snai1 and silencing fibroblast signatures. EMBO J.

[b118] Addis RC, Ifkovits JL, Pinto F (2013). Optimization of direct fibroblast reprogramming to cardiomyocytes using calcium activity as a functional measure of success. J Mol Cell Cardiol.

[b119] Ifkovits JL, Addis RC, Epstein JA (2014). Inhibition of TGFbeta signaling increases direct conversion of fibroblasts to induced cardiomyocytes. PLoS ONE.

[b120] Wang L, Liu Z, Yin C (2015). Stoichiometry of Gata4, Mef2c, and Tbx5 influences the efficiency and quality of induced cardiac myocyte reprogramming. Circ Res.

[b121] Wang H, Cao N, Spencer CI (2014). Small molecules enable cardiac reprogramming of mouse fibroblasts with a single factor, Oct4. Cell Rep.

[b122] Qian L, Huang Y, Spencer CI (2012). *In vivo* reprogramming of murine cardiac fibroblasts into induced cardiomyocytes. Nature.

[b123] Inagawa K, Miyamoto K, Yamakawa H (2012). Induction of cardiomyocyte-like cells in infarct hearts by gene transfer of Gata4, Mef2c, and Tbx5. Circ Res.

[b124] Jayawardena TM, Finch EA, Zhang L (2015). MicroRNA induced cardiac reprogramming *in vivo*: evidence for mature cardiac myocytes and improved cardiac function. Circ Res.

[b125] Nam Y-J, Song K, Luo X (2013). Reprogramming of human fibroblasts toward a cardiac fate. Proc Natl Acad Sci USA.

[b126] Fu JD, Stone NR, Liu L (2013). Direct reprogramming of human fibroblasts toward a cardiomyocyte-like state. Stem Cell Reports.

[b127] Wada R, Muraoka N, Inagawa K (2013). Induction of human cardiomyocyte-like cells from fibroblasts by defined factors. Proc Natl Acad Sci USA.

[b128] Porrello ER, Mahmoud AI, Simpson E (2011). Transient regenerative potential of the neonatal mouse heart. Science.

[b129] Porrello ER, Mahmoud AI, Simpson E (2013). Regulation of neonatal and adult mammalian heart regeneration by the miR-15 family. Proc Natl Acad Sci USA.

[b130] Xie M, Cao N, Ding S (2014). Small molecules for cell reprogramming and heart repair: progress and perspective. ACS Chem Biol.

[b131] Kuhn B, del Monte F, Hajjar RJ (2007). Periostin induces proliferation of differentiated cardiomyocytes and promotes cardiac repair. Nat Med.

[b132] Polizzotti BD, Arab S, Kühn B (2012). Intrapericardial delivery of gelfoam enables the targeted delivery of periostin peptide after myocardial infarction by inducing fibrin clot formation. PLoS ONE.

[b133] Ladage D, Yaniz-Galende E, Rapti K (2013). Stimulating myocardial regeneration with periostin peptide in large mammals improves function post-myocardial infarction but increases myocardial fibrosis. PLoS ONE.

[b134] Lorts A, Schwanekamp JA, Elrod JW (2009). Genetic manipulation of periostin expression in the heart does not affect myocyte content, cell cycle activity, or cardiac repair. Circ Res.

[b135] P Blomberg C, Lee J, P Morgan J (2014). The developing role of Neuregulin1 in cardiac regenerative stem cell therapy. Curr Pharm Des.

[b136] Bersell K, Arab S, Haring B (2009). Neuregulin1/ErbB4 signaling induces cardiomyocyte proliferation and repair of heart injury. Cell.

[b137] Polizzotti BD, Ganapathy B, Walsh S (2015). Neuregulin stimulation of cardiomyocyte regeneration in mice and human myocardium reveals a therapeutic window. Sci Transl Med.

[b138] D’Uva G, Aharonov A, Lauriola M (2015). ERBB2 triggers mammalian heart regeneration by promoting cardiomyocyte dedifferentiation and proliferation. Nat Cell Biol.

[b139] Mendes-Ferreira P, De Keulenaer GW, Leite-Moreira AF (2013). Therapeutic potential of neuregulin-1 in cardiovascular disease. Drug Discovery Today.

[b140] Reiss K, Cheng W, Ferber A (1996). Overexpression of insulin-like growth factor-1 in the heart is coupled with myocyte proliferation in transgenic mice. Proc Natl Acad Sci USA.

[b141] Naqvi N, Li M, Calvert John W (2014). A proliferative burst during preadolescence establishes the final cardiomyocyte number. Cell.

[b142] Engel FB, Hsieh PC, Lee RT (2006). FGF1/p38 MAP kinase inhibitor therapy induces cardiomyocyte mitosis, reduces scarring, and rescues function after myocardial infarction. Proc Natl Acad Sci USA.

[b143] Choi WY, Gemberling M, Wang J (2013). *In vivo* monitoring of cardiomyocyte proliferation to identify chemical modifiers of heart regeneration. Development.

[b144] Mahmoud AI, Kocabas F, Muralidhar SA (2013). Meis1 regulates postnatal cardiomyocyte cell cycle arrest. Nature.

[b145] Cheng RK, Asai T, Tang H (2007). Cyclin A2 induces cardiac regeneration after myocardial infarction and prevents heart failure. Circ Res.

[b146] Shapiro SD, Ranjan AK, Kawase Y (2014). Cyclin A2 induces cardiac regeneration after myocardial infarction through cytokinesis of adult cardiomyocytes. Sci Transl Med.

[b147] Eulalio A, Mano M, Ferro MD (2012). Functional screening identifies miRNAs inducing cardiac regeneration. Nature.

[b148] Tian Y, Liu Y, Wang T (2015). A microRNA-Hippo pathway that promotes cardiomyocyte proliferation and cardiac regeneration in mice. Sci Transl Med.

[b149] Heallen T, Morikawa Y, Leach J (2013). Hippo signaling impedes adult heart regeneration. Development.

[b150] Lin Z, von Gise A, Zhou P (2014). Cardiac-specific YAP activation improves cardiac function and survival in an experimental murine MI model. Circ Res.

[b151] Lin Z, Pu WT (2015). Releasing YAP from an alpha-catenin trap increases cardiomyocyte proliferation. Circ Res.

[b152] Xin M, Kim Y, Sutherland LB (2013). Hippo pathway effector Yap promotes cardiac regeneration. Proc Natl Acad Sci USA.

[b153] Hodgetts SI, Beilharz MW, Scalzo AA (2000). Why do cultured transplanted myoblasts die *in vivo*? DNA quantification shows enhanced survival of donor male myoblasts in host mice depleted of CD4^+^ and CD8^+^ cells or Nk1.1^+^ cells. Cell Transplant.

[b154] Bayomy AF, Bauer M, Qiu Y (2012). Regeneration in heart disease-Is ECM the key?. Life Sci.

[b155] Pagani FD, DerSimonian H, Zawadzka A (2003). Autologous skeletal myoblasts transplanted to ischemia-damaged myocardium in humans. Histological analysis of cell survival and differentiation. J Am Coll Cardiol.

[b156] Haider H, Ashraf M (2008). Strategies to promote donor cell survival: combining preconditioning approach with stem cell transplantation. J Mol Cell Cardiol.

[b157] Davis ME, Hsieh PC, Takahashi T (2006). Local myocardial insulin-like growth factor 1 (IGF-1) delivery with biotinylated peptide nanofibers improves cell therapy for myocardial infarction. Proc Natl Acad Sci USA.

[b158] Schofield R (1978). The relationship between the spleen colony-forming cell and the haemopoietic stem cell. Blood cells.

[b159] Scadden DT (2006). The stem-cell niche as an entity of action. Nature.

[b160] Bani D, Nistri S (2014). New insights into the morphogenic role of stromal cells and their relevance for regenerative medicine. lessons from the heart. J Cell Mol Med.

[b161] Hatzistergos KE, Quevedo H, Oskouei BN (2010). Bone marrow mesenchymal stem cells stimulate cardiac stem cell proliferation and differentiation. Circ Res.

[b162] Guarita-Souza LC, Carvalho KA, Woitowicz V (2006). Simultaneous autologous transplantation of cocultured mesenchymal stem cells and skeletal myoblasts improves ventricular function in a murine model of Chagas disease. Circulation.

[b163] Melero-Martin JM, De Obaldia ME, Kang SY (2008). Engineering robust and functional vascular networks *in vivo* with human adult and cord blood-derived progenitor cells. Circ Res.

[b164] Gherghiceanu M, Popescu LM (2012). Cardiac telocytes - their junctions and functional implications. Cell Tissue Res.

[b165] Popescu LM, Curici A, Wang E (2015). Telocytes and putative stem cells in ageing human heart. J Cell Mol Med.

[b166] Fertig ET, Gherghiceanu M, Popescu LM (2014). Extracellular vesicles release by cardiac telocytes: electron microscopy and electron tomography. J Cell Mol Med.

[b167] Cismasiu VB, Popescu LM (2015). Telocytes transfer extracellular vesicles loaded with microRNAs to stem cells. J Cell Mol Med.

[b168] Bei Y, Zhou Q, Fu S (2015). Cardiac telocytes and fibroblasts in primary culture: different morphologies and immunophenotypes. PLoS ONE.

[b169] Zhao B, Chen S, Liu J (2013). Cardiac telocytes were decreased during myocardial infarction and their therapeutic effects for ischaemic heart in rat. J Cell Mol Med.

[b170] Zhao B, Liao Z, Chen S (2014). Intramyocardial transplantation of cardiac telocytes decreases myocardial infarction and improves post-infarcted cardiac function in rats. J Cell Mol Med.

[b171] Williams AR, Hatzistergos KE, Addicott B (2013). Enhanced effect of combining human cardiac stem cells and bone marrow mesenchymal stem cells to reduce infarct size and to restore cardiac function after myocardial infarction. Circulation.

[b172] Katare R, Riu F, Mitchell K (2011). Transplantation of human pericyte progenitor cells improves the repair of infarcted heart through activation of an angiogenic program involving micro-RNA-132. Circ Res.

[b173] Avolio E, Meloni M, Spencer HL (2015). Combined intramyocardial delivery of human pericytes and cardiac stem cells additively improves the healing of mouse infarcted hearts through stimulation of vascular and muscular repair. Circ Res.

[b174] Ahmadi A, McNeill B, Vulesevic B (2014). The role of integrin alpha2 in cell and matrix therapy that improves perfusion, viability and function of infarcted myocardium. Biomaterials.

[b175] Christalla P, Hudson JE, Zimmermann WH (2012). The cardiogenic niche as a fundamental building block of engineered myocardium. Cells Tissues Organs.

[b176] Mayfield AE, Tilokee EL, Latham N (2014). The effect of encapsulation of cardiac stem cells within matrix-enriched hydrogel capsules on cell survival, post-ischemic cell retention and cardiac function. Biomaterials.

[b177] Forbes SJ, Rosenthal N (2014). Preparing the ground for tissue regeneration: from mechanism to therapy. Nat Med.

[b178] Pascual-Gil S, Garbayo E, Diaz-Herraez P (2015). Heart regeneration after myocardial infarction using synthetic biomaterials. J Controlled Release.

[b179] Zhang S, Ma X, Yao K (2014). Combination of CD34-positive cell subsets with infarcted myocardium-like matrix stiffness: a potential solution to cell-based cardiac repair. J Cell Mol Med.

[b180] Dai W, Hale SL, Kay GL (2009). Delivering stem cells to the heart in a collagen matrix reduces relocation of cells to other organs as assessed by nanoparticle technology. Regen Med.

[b181] Emmert MY, Hitchcock RW, Hoerstrup SP (2014). Cell therapy, 3D culture systems and tissue engineering for cardiac regeneration. Adv Drug Deliv Rev.

[b182] Rojas SV, Martens A, Zweigerdt R (2015). Transplantation effectiveness of induced pluripotent stem cells is improved by a fibrinogen biomatrix in an experimental model of ischemic heart failure. Tissue Eng Part A.

[b183] Singelyn JM, Christman KL (2010). Injectable materials for the treatment of myocardial infarction and heart failure: the promise of decellularized matrices. Cardiovasc Transl Res.

[b184] Oberwallner B, Brodarac A, Anic P (2015). Human cardiac extracellular matrix supports myocardial lineage commitment of pluripotent stem cells. Eur J Cardiothorac Surg.

[b185] Wang H, Shi J, Wang Y (2014). Promotion of cardiac differentiation of brown adipose derived stem cells by chitosan hydrogel for repair after myocardial infarction. Biomaterials.

[b186] Hamdi H, Planat-Benard V, Bel A (2014). Long-term functional benefits of epicardial patches as cell carriers. Cell Transplant.

[b187] Savi M, Bocchi L, Fiumana E (2015). Enhanced engraftment and repairing ability of human adipose-derived stem cells, conveyed by pharmacologically active microcarriers continuously releasing HGF and IGF-1, in healing myocardial infarction in rats. J Biomed Mater Res Part A.

[b188] Lin YD, Yeh ML, Yang YJ (2010). Intramyocardial peptide nanofiber injection improves postinfarction ventricular remodeling and efficacy of bone marrow cell therapy in pigs. Circulation.

[b189] Ban K, Park HJ, Kim S (2014). Cell therapy with embryonic stem cell-derived cardiomyocytes encapsulated in injectable nanomatrix gel enhances cell engraftment and promotes cardiac repair. ACS Nano.

[b190] Lin YD, Chang MY, Cheng B (2015). Injection of peptide nanogels preserves postinfarct diastolic function and prolongs efficacy of cell therapy in pigs. Tissue Eng Part A.

[b191] ClinicalTrials.gov (2015). http://clinicaltrials.gov/ct2/show/NCT01458405.

[b192] ClinicalTrials.gov (2015). http://clinicaltrials.gov/ct2/show/NCT01508910.

[b193] ClinicalTrials.gov (2015). http://clinicaltrials.gov/ct2/show/NCT01495364.

[b194] ClinicalTrials.gov (2015). http://clinicaltrials.gov/ct2/show/NCT01781390.

[b195] ClinicalTrials.gov (2015). http://clinicaltrials.gov/ct2/show/NCT01216995.

[b196] ClinicalTrials.gov (2015). http://clinicaltrials.gov/ct2/show/NCT01449032.

[b197] ClinicalTrials.gov (2015). http://clinicaltrials.gov/ct2/show/NCT01556022.

[b198] ClinicalTrials.gov (2015). http://clinicaltrials.gov/ct2/show/NCT02052427.

[b199] ClinicalTrials.gov (2015). http://clinicaltrials.gov/ct2/show/NCT01770613.

[b200] ClinicalTrials.gov (2015). http://clinicaltrials.gov/ct2/show/NCT02123706.

[b201] ClinicalTrials.gov (2015). http://clinicaltrials.gov/ct2/show/NCT00877903.

